# Death from Intra-Uterine Injection of Glycerin

**Published:** 1899-09

**Authors:** 


### DEATH FROM INTRA-UTERINE INJECTION OF GLYCERIN.

The patient was a woman, about thirty years of age, and pregnant for the second time. At about the second month of gestation she resolved to terminate the pregnancy by the intra-uterine injection of glycerin. The quantity of glycerin used is uncertain, but it is said to have been but a few drams. This was introduced into the uterus by means of an English catheter, with a syringe attached. The ovum was expelled on the following day. Within twenty-four hours after the injection she was seized with moderate chills, and the temperature rose to 103 degrees F.; for the next two days it ranged between 103 degrees and 104 degrees F. From the third day the temperature did not exceed 99 degrees, and for the most of the time was subnormal. After the second day there were frequent vomiting and diarrhœa. On the third the teeth were covered with sordes, the saliva was blood-stained, and a profuse hemorrhage occurred from the nose, which was controlled only by plugging. The vomitus consisted of coffee-ground material, and later the stools were tarry. The urine was from the first almost wholly suppressed, and was of a dark-red color from the presence of hæmaglobin. Death occurred on the sixth day.

The dangers of Pelzer's method are well-known, and they have led to its abandonment. Luchsinger, Schwan, Filehne, Lébédeff, and Wiener have shown that glycerin is liable to cause disintegration of the red blood cells. The destructive action of the glycerin upon the red blood corpuscles obviously cannot be prevented by any care that can be used in the intra-uterine injection. Affanassiew's experiments would seem to prove that the blood is more surely disorganized when the drug is absorbed through the decidua than if thrown into an open sinus.—Dr. Charles Jewett, Brooklyn.—*Modern Medical Science,* July, 1899.

